# Duodenal Diverticulitis: To Operate or Not To Operate?

**DOI:** 10.7759/cureus.6236

**Published:** 2019-11-26

**Authors:** Sahand Bamarni, Suysen Hung Fong, Dereen Mohammed Saeed, Subhasis Misra, In Soon Park

**Affiliations:** 1 Surgery, Brandon Regional Hospital, Brandon, USA; 2 Pathology, University of Illinois, Chicago, USA

**Keywords:** duodenal diverticulum, duodenal diverticulitis, duodenum

## Abstract

Duodenal diverticulum (DD) is a common incidental finding, which rarely causes complications. Perforation is one of the most feared and the least common complications. Surgery is the mainstay for complicated duodenal diverticulum, but with the advancement of medical treatment and intensive care, nonoperative management has been reported. We present a rare case of perforated DD that failed medical management and subsequently underwent surgical intervention.

A 77-year-old, healthy female presented with right-sided abdominal pain with low-grade fever and leukocytosis. Computed tomography (CT) of the abdomen showed retroperitoneal fluid collection around the second part of the duodenum, which was not amenable to percutaneous drainage. Contrast studies showed no evidence of perforation or leak of the stomach or duodenum. The diagnosis was made via an upper endoscopy that showed a large periampullary duodenal diverticulum with purulent drainage and normal-looking ampulla. After failed conservative management with broad-spectrum antibiotics and worsening symptoms, she underwent excision and primary repair of the diverticulum with a jejunal serosal patch and exploration of the common bile duct (CBD). She had an uncomplicated postoperative course and was discharged home on postoperative day four.

Although rare, the duodenal diverticular perforation can be a life-threatening complication. Combined subjective, clinical, and radiological assessment of the patient is crucial in deciding whether to operate or not.

## Introduction

The duodenum is the second most common site for diverticula following the colon [1]. The incidence of duodenal diverticula (DD) is estimated to be 22% [1]. It is usually located near the papilla of Vater [1]. DD could be congenital or acquired; most are acquired and extraluminal, as this is secondary to the protrusion of an outpouching near the entrance of a large vessel while congenital diverticula are usually intraluminal and develop secondary to incomplete canalization [1]. DD is often found incidentally on upper gastrointestinal contrast study or autopsy [2]. The majority of the DD are asymptomatic or may present with nonspecific symptoms of abdominal pain, nausea, or vomiting and fever [2]. DD may present with serious complications like perforation, duodenal fistulas, intra-abdominal abscesses, and sepsis [1-2]. Historically, surgery was the main modality of management, but with recent advances in medical treatment, nonoperative management has been reported to be successful in multiple cases [3]. Surgical intervention is reserved for patients who develop complications associated with diverticulitis such as bowel perforation, abscess, or fistula [2].

## Case presentation

**Figure 1:**
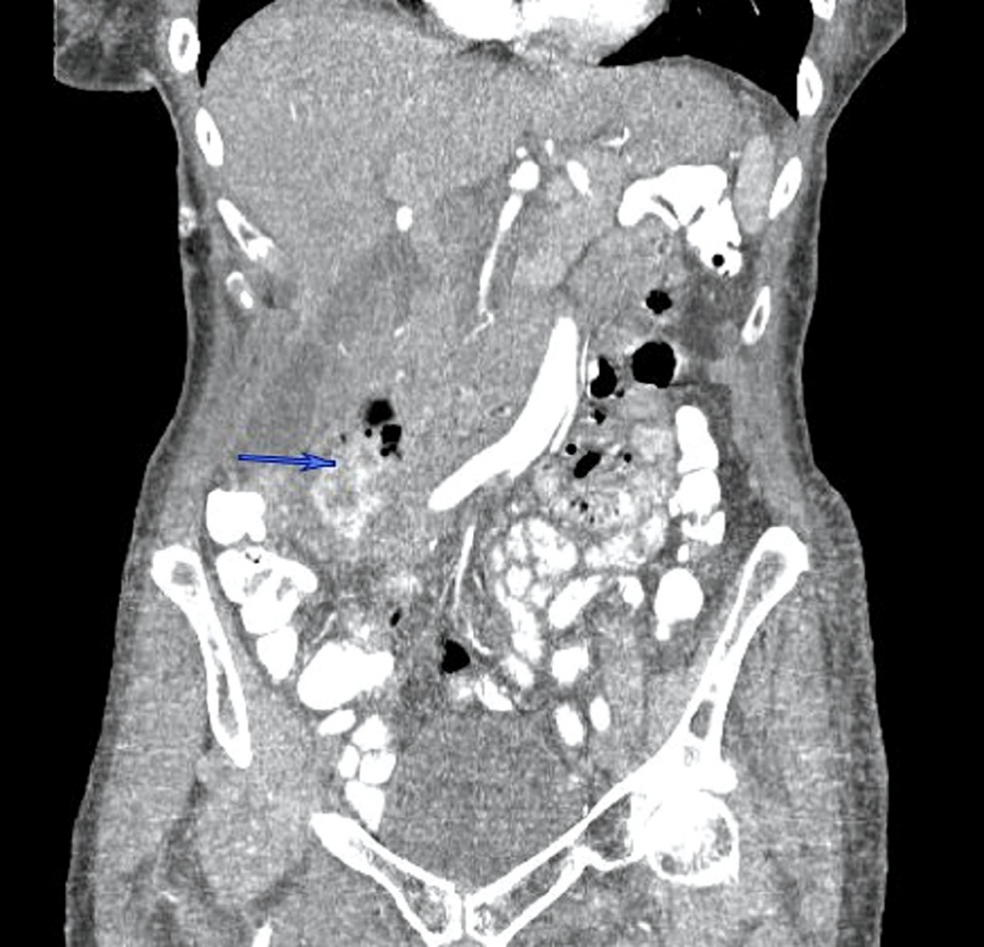
CT abdomen (coronal section) demonstrates a complex collection with a contained contrast leak related to the perforated duodenal diverticulum

**Figure 2:**
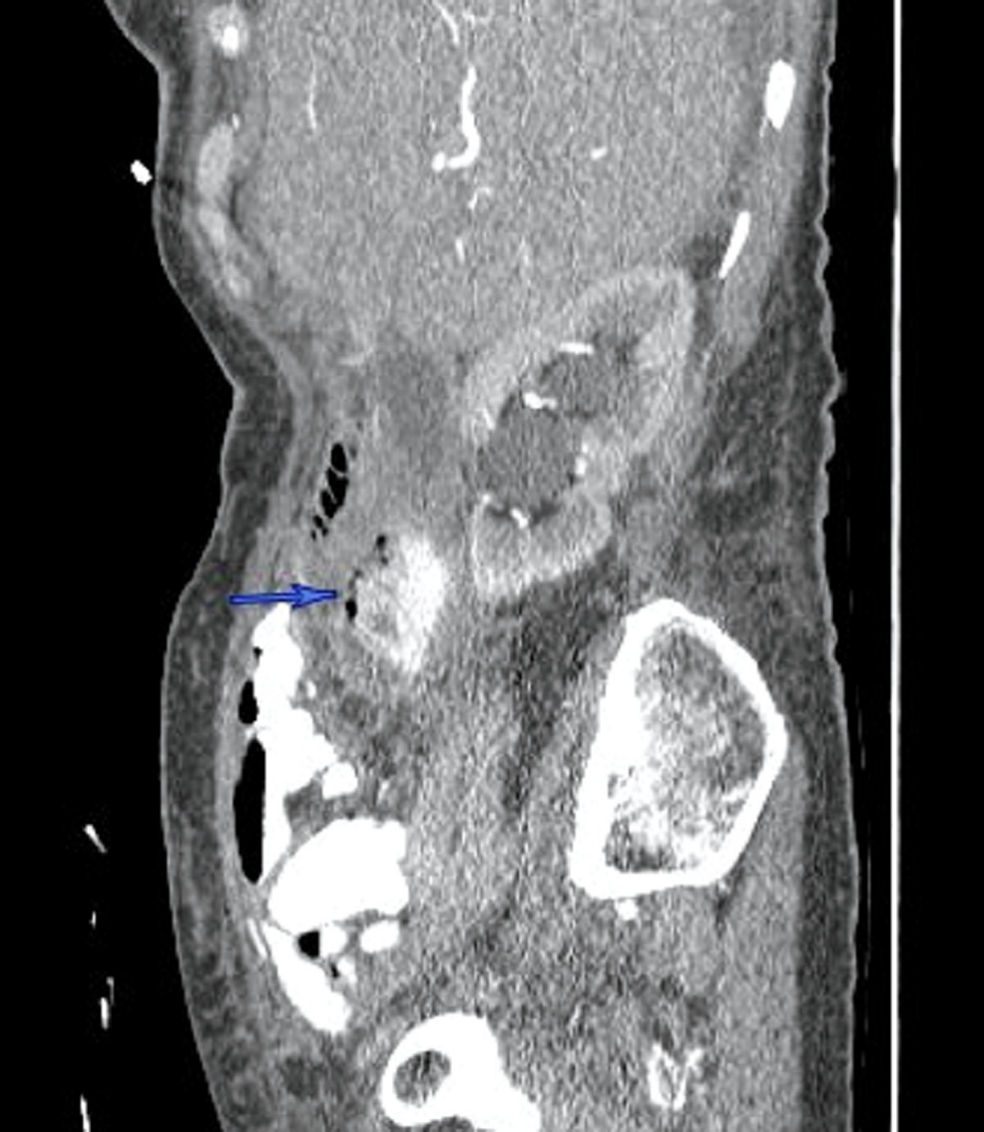
CT abdomen (sagittal section) demonstrates a complex collection with a contained contrast leak related to the perforated duodenal diverticulum

A 77-year-old healthy female presented with right-sided abdominal pain, associated with low-grade fever to 38.2 °C and mild epigastric tenderness. She was hemodynamically stable, with no significant past medical or surgical history. An outpatient CT of the abdomen (Figures 1-2) ordered by her primary care physician (PCP) revealed retroperitoneal fluid collection around the second part of the duodenum, which prompted her to be admitted to the hospital. She was found to have a low-grade fever with leukocytosis, and broad-spectrum antibiotics were immediately started. After a review of the CT images of the abdomen with the interventional radiologist, the retroperitoneal fluid collection was determined to be not amenable to percutaneous drainage. An upper gastrointestinal (GI) and small bowel study showed no evidence of perforation or leak of the stomach or duodenum. Subsequently, an upper endoscopy showed a large periampullary duodenal diverticulum with purulent drainage and normal-looking ampulla. After 72 hours of conservative management with NPO, intravenous (IV) fluid, and antibiotics, the decision was made to proceed with surgery due to persistent epigastric pain and tenderness with an interval increase in the retroperitoneal collection. She underwent excision and primary repair of the diverticulum with a jejunal serosal patch and exploration of the common bile duct (CBD) due to the proximity of the diverticulum to the ampulla. She had an uncomplicated postoperative course and was discharged home on the fourth postoperative day.

## Discussion

There are two types of DD, congenital and acquired. The majority of the diverticula are acquired extraluminal pseudodiverticula [3]. DD commonly arises from the second part of the duodenum, within 2 cm of the ampulla of Vater due to potential wall weakness at the papilla [3]. Although DD is asymptomatic in 90% of the cases, it may present with various complications [3]. The most common complications of DD are biliopancreatic stasis and obstruction. Other complications include ulceration with bleeding and diverticulitis with a possible perforation. The perforation is often associated with diverticulitis or ischemia due to distention from food retention inside the diverticulum. Other causes of perforation include ulceration, iatrogenic trauma, and foreign bodies [4]. The retroperitoneal perforation of DD is usually contained and presents with no signs of peritoneal irritation. The patient typically will present with upper abdominal pain associated with nausea and vomiting. Cholestasis and elevated lipase may be noted with inflammation and compression effects.

Historically, perforated DD was treated surgically, but most recently, several reports have shown good outcomes with nonoperative management [2-3]. The overall patient clinical presentation and hemodynamic stability should guide the mode of management and be tailored on a case-by-case basis.

Surgical management is challenging due to the proximity of the diverticulum to the papilla; hence, it is highly recommended to identify the papilla before surgery preferably via an upper endoscopy or intraoperatively by inserting a catheter by cholecystectomy or choledochotomy [5-6]. Patients with stable vitals and without signs of peritonitis should begin with nonoperative management. Although the standard treatment of DD was suggested to be surgical, given the high rate of complications associated with surgery, including duodenal leak, fistula, and sepsis, surgical intervention is warranted only in complicated cases [1,7].

A recent case series by Thorson et al. showed that nonoperative management carries a lower morbidity and mortality rate than an operative approach [8]. A classical surgical intervention of DD includes diverticulectomy with double layer closure [8-9]. More complex interventions are required for those with extensive retroperitoneal inflammation, such as pyloric exclusion, gastroduodenostomy, or gastrojejunostomy; duodenostomy and pylorus-preserving Whipple might be indicated [9-10].

## Conclusions

We report a case of complicated DD who failed nonoperative management and subsequently underwent surgery. DD are often asymptomatic but may present with perforation with subsequent retroperitoneal inflammation and infection. Nonoperative management should be attempted for a clinically stable non-peritonitic patient. Surgery is a challenging approach given their location and close proximity to the ampulla of Vater. Various surgical approaches could be performed depending on the individual clinical status.
